# Masters or pawns? Examining injury and chronic disease in male Masters Athletes and chess players compared to population norms from the Canadian Community Health Survey

**DOI:** 10.1186/s11556-018-0204-z

**Published:** 2018-11-30

**Authors:** Shruti Patelia, Rachael C. Stone, Rona El-Bakri, Mehrnaz Adli, Joseph Baker

**Affiliations:** 1 School of Kinesiology and Health Science, York University, 116 Norman Bethune College, 4700 Keele St., Toronto, ON M3J 1P3 Canada; 2 Queen’s University, 301N Kinesiology and Health Studies Building, 99 University Ave, Kingston, ON K7L 3N6 Canada

**Keywords:** Masters athletes, Chess players, Injury, Chronic disease & older adults

## Abstract

**Background:**

Identifying the optimal type and amount of activity for the maintenance of function in older adults has proved challenging. On the one hand, Masters Athletes have been proposed as the ideal model of successful aging but most of this research has focused on physical functioning. On the other hand, the importance of cognitive engagement has been emphasized, which may be more strongly related to activities such as playing chess. The current study aimed to compare physical health outcomes (i.e., prevalence of physical injury and chronic disease) among older athletes and chess players. Masters Athletes and chess players were recruited from track and field and chess competitions within the province of Ontario. In addition to these primary groups, moderately active and inactive older adults from Canadian Community Health Survey were also included for comparison.

**Results:**

Masters Athletes had significantly higher rates of injury with the lowest rates of chronic disease, compared to all other activity groups. In contrast, chess players reported lower rates of injury compared to Masters Athletes as well as lower prevalence of chronic diseases compared to the moderately active and inactive groups. The normative groups reported the lowest rate of injury, but increased prevalence of chronic diseases compared to Masters Athletes and chess players.

**Conclusions:**

Findings from this study indicate that both athletic and cognitive engagement may be positively related to the physical health of older adults, since Masters Athletes and chess players reported a lower prevalence of chronic disease. Importantly, the results expand our current understanding of health by providing evidence for physical health outcomes associated with an activity that is primarily associated with cognitive health.

## Introduction

Many older adults have an increased likelihood of living longer with multiple comorbidities (e.g., arthritis, high blood pressure, injury) that can require continual financial and medical care [1–3]. Additionally, because many health care systems were designed to provide acute, episodic care for a relatively young population, these systems are ill equipped to manage the complex health care needs of this population (~ 75–80% reporting one or more chronic diseases) [4–6]. Fortunately, modifiable lifestyle factors such as participating in physically and cognitively challenging activities can be useful for mitigating and managing many health-related issues among older adults [3, 7]. Engagement in diverse forms of activities has been shown to provide a wide range of physical [8, 9], cognitive [10, 11] and psychosocial benefits [12–15]. Different types of activities may also provide different health benefits, which can be crucial in maintaining overall well-being to age successfully [16–19].

Interestingly, while there is considerable evidence emphasizing the benefits of maintaining an active lifestyle irrespective of age [7, 20], most studies of older adults have focused on activities of low to moderate exercise intensity (i.e., walking, dancing, fitness classes) [14], and, as a result, we have little information on the health outcomes associated with older adults who regularly participate in more intensive activities such as competitive sport [21, 22]. However, the growing number of baby boomers competing in sport has recently caught the attention of researchers interested in optimizing the health and function of older adults [23, 24]. Moreover, there is increasing momentum for participation in competitive sport. For example, participation in the World Masters Games has increased from 8305 competitors in 1985 to 28,905 in 2017 [25]. In Canada, a 2 to 3-fold increase has been observed in the number of sport participants aged 55 and above [26]. This proliferation has motivated researchers to examine *Masters Athletes,* defined as individuals generally above the age of 35 who continually maintain a high intensity of exercise by competing in Master sport [23].

Despite having little information on the health and functional benefits of competitive sport [22, 24], some researchers have proposed older (i.e., Masters) athletes as the ideal model of successful aging [27]. A large body of evidence with this group has focused on areas such as maintaining performance despite declines in physical function or modulating the factors responsible for age-related decline [28–31]. Interestingly, research on the health and function of Masters Athletes, suggest continual participation in sport is associated with lower risk of certain chronic diseases (e.g., chronic chest pain, asthma, heart attack and diabetes) [12, 32]. In addition, while there is some information on sport-related injury among older athletes [26, 31, 33–35], little is known on the prevalence of injury in older Masters Athletes in comparison to non-athletes [36, 37]. This could be important, since regular sport participation can accelerate the onset of conditions such as osteoarthritis as a result of overuse [38], whereas some chronic diseases (e.g., osteoporosis) can increase the risk of severe injury [32, 39].

Furthermore, the literature on healthy aging in older adults has primarily focused on the benefits of physical engagement, with much less attention dedicated to other types of engagement [40]. This is surprising since physical activity (i.e., sport) represents only one of the many types of active engagement older adults may experience and other types of activities may be beneficial for maintaining other components of health [13, 40]. For example, previous research suggests cognitive engagement has many associated mental health benefits (e.g., improvement of memory or delaying dementia) and is useful for maintaining cognitive function [11, 41–43]. This type of active engagement is especially important to help manage the 66% rise in older adults with dementia expected to occur in the next few years [2]. Furthermore, in relation to physical health, previous studies have found older adults with some chronic diseases (e.g., hypertension, heart disease, chronic obstructive pulmonary disorder) score significantly lower on cognitive performance tasks (e.g., block design, object assembly, word recall, visuospatial tests) compared to older adults without these diseases [44–46].

Interestingly, none of the studies of Masters Athletes has considered them relative to other forms of engagement. To this end, we compared the physical health of older adults, who participated in competitive sport with similar aged older adults (i.e., 50 years and above) who competed in chess competitions during the time of data collection. This activity was chosen because of its popularity, objective performance measure (i.e., Elo score), and the intense practice required to become an expert competitive participant [47, 48]. This allows for an intriguing comparison between older adults who are highly physically active with those who are highly cognitively active. Moreover, exploring competitive chess may increase our understanding on the health outcomes associated with older adults who are actively engaged in a sedentary activity. Based on limited past research [32, 49], we hypothesized Master Athletes would have the highest rates of injury and the lowest prevalence of chronic disease when compared to chess players and normative groups, due to their continual participation in vigorous activity.

## Methods

### Study design

A cross-sectional between subjects design was used to compare Masters Athletes, and chess players. In addition, these two primary groups were compared to normative data representing two groups: moderately active and inactive older adults. These data were drawn from the Canadian Community Health Survey (CCHS) cycle 4.1, a large-scale National survey that is representative of the Canadian population [50]^1^ Masters Athletes and chess players were recruited from local track and field and chess competitions organized by Ontario Masters Athletics and The Chess Federation of Canada, respectively. Electronic versions of the questionnaire were also provided by email and made available on their websites. Researchers from this study obtained informed consent from all athletes and chess players prior to completing the survey and this project received institutional ethics approval (e2015–232).

### Participants

Following ethics approval, researchers contacted various Masters and chess clubs, events and competitions taking place in Ontario. Over a 5-month period, a total of 146 Masters track and field athletes were recruited from 9 clubs and competitions that agreed to participate in the study (i.e., Taylor Creek 5 K Cross Country, Don Farquharson Harriers 4 k, Sunnybrook 8 k, Ontario Cross Country Championships, Canadian Cross Country Championships, New Market Huskies, University of Toronto Track, Longboat runners, and Black Lungs Toronto). Concurrently, a total of 68 chess players were recruited from 6 clubs and competitions (i.e., Harbourfront Centre Chess-fest competition, Kitchener-Waterloo Chess competition, Annex Chess, Kingsview Chess, Willowdale Chess, and Chess Institute of Canada). After limiting the sample to those who were aged 50 and above, completed the injury and chronic disease section, and male,^2^ a total of 50 chess players and 69 Masters Athletes were included in the final analyses. Similarly, after following the same filtering procedure as well as case-matching on socio-demographic variables with Masters Athletes, a total of 64 moderately active and 62 inactive older adults were randomly selected from the CCHS dataset for inclusion in the secondary comparison.

### Measures

In order to facilitate comparisons between our primary groups of interest (i.e., Masters Athletes and chess player) and the normative groups selected from the CCHS (i.e., moderately active and inactive), all questions were drawn from the CCHS dataset. Each question is described below.

#### Injury

Prevalence of injury was defined and measured using the following questions: *In the past 12 months, did you experience an injury which limited your normal activities? If “yes” did you sustained “1”, “2”, “3 to 5”, or “6 or more injuries”* [50]. To verify that reported injuries corresponded with the number of injuries reported and were acute in nature (e.g., participants with repetitive knee pain would be removed), responses were compared with another question regarding type of injuries [50]. As a result, participants were removed if there were any discrepancies between the number of injuries and types of injuries sustained in the past 12 months (e.g., if they reported 3 to 5 injuries in total but 7 sprain and strain injuries).

#### Chronic disease

The presence of chronic disease was defined and measured using the following question: *In the past 6 months or more, were you diagnosed by a health professional with a type of chronic disease? If yes, please specify the type of chronic disease* [50]. The original scoring of this question used “yes = 1” and “no = 2” and in the current analyses all sixteen chronic diseases questions were combined into a total score, where lower scores reflected greater prevalence of chronic disease (e.g., a score of 32 would reflect the sum of all “no = 2” and therefore equal “no chronic disease”, 31 = “one chronic disease” and so on). Due to the distribution of chronic disease across our samples and to simplify the reporting of our analyses, respondents were coded as either zero, one, two, three, or four or more chronic diseases.

#### Activity type

The *physical activity index* was selected from the CCHS 4.1 because it had a clear definition for active, moderately active and inactive older adults according to average daily energy expenditure (EE).^3^ To this end, ‘active’ participants expended > 2.9 kcal/kg/d, ‘moderately active’ expended between 1.5 to < 3 kcal/kg/d and ‘inactive’ expended < 1.5 kcal/kg/d [50]. In order to compare the normative groups with Masters Athletes, the ‘active group’ (i.e., > 2.9 kcal/kg/d) from the CCHS 4.1 was removed to ensure no overlap between those reporting high levels of physical activity and exercise (which may have included older sport participants) and our Masters athlete group. Similarly, chess players who expended > 2.9 kcal/kg/d were removed from the dataset. Finally, in line with previous research, this study accounted for possible socio-demographic variables that may pose as confounders such as marital status, income, education, and age [51].

### Analyses

A multi-pronged approach was considered due to the increased variability between the activity groups. Preliminary normality analyses from the Shapiro-Wilks tests, suggested Masters athletes and chess players were not normality distributed in their demographics (i.e., age and income) compared to the normative groups. As a result, Kruskal-Wallis (K-W) non-parametric tests were performed to compare mean ranks among activity groups; however, non-parametric tests can be problematic when variability differs between groups (e.g., sport, physical activity and chess). As a result, K-W tests were followed by analysis of covariance (ANCOVA) tests using Bonferroni post hoc tests with an adjusted alpha of 0.0125. Pre-analyses (see Table 1) indicated the athletes and chess players were predominantly mid-aged (50–59 years), affluent (≥ $80,000), married/domestic relationship and educated (post-secondary graduate). In an attempt to create more homogeneous groups for comparisons, moderately active and inactive groups were case-matched on age and income with the Masters Athletes and then randomly selected from the larger group to create analogous sample sizes. To account for possible variability that may still exist between chess players and normative groups, socio-demographic variables were included as covariates in the ANCOVA analyses. Repeating the non-parametric and parametric tests on the case-matched dataset helped eliminate potential confounders, increase validity, and gain coherence between groups. Finally, Chi-square analyses were performed to compare types of injuries and chronic diseases reported by the activity groups.

## Results

Descriptively, the Masters Athletes reported the highest prevalence (69.6%) of injuries compared to chess players (24%), moderately active (14.1%) and inactive adults (14.5%). They also reported the lowest prevalence of chronic diseases (15.9%), followed by the chess players (30%), inactive (64.5%) and moderately active (67.2%) older adults (Table 1).

**Table 1:** Background information on athletes, chess players, moderately and inactive older adults (*N* = 245)

	Masters Athletes	Chess	Moderately active	Inactive
Age				
50–59 years	33 (49%)	20 (40%)	27 (42%)	28 (45%)
60–69 years	21 (30.4%)	17 (34%)	21 (32.8%)	21 (33.9%)
70–79 years	10 (14.5%)	8 (16%)	10 (15.6%)	9 (14.5%)
80 and above	5 (7.2%)	5 (10%)	6 (9.4%)	4 (6.5%)
Marital Status				
Married/Domestic relationship	33 (47.8%)	30 (60%)	48 (75%)	44 (71%)
Divorced/Widowed/Separated	9 (13.0%)	11 (22%)	12 (18.8%)	12 (19.4%)
Single/Never married	27 (39.1%)	9 (18%)	4 (6.3%)	6 (9.7%)
Income				
<$60,000	15 (21.7%)	22 (44%)	11 (17.2%)	9 (14.5%)
$60,000 - $79,000	6 (8.7%)	4 (8%)	6 (9.4%)	8 (12.9%)
$80,000 or more	48 (69.6%)	24 (48%)	47 (73.4%)	45 (72.6%)
Education				
< Secondary school	6 (8.7%)	3 (6%)	9 (14.1%)	11 (17.7%)
Secondary graduate	6 (8.7%)	5 (10%)	8 (12.5%)	14 (22.6%)
Other post-secondary	9 (13.0%)	14 (28%)	3 (4.7%)	3 (4.8%)
Post-secondary graduate	48 (69.6%)	28 (56%)	44 (68.8%)	34 (54.8%)
Years competing				
< 5 years	19 (27.9%)	2 (4.4%)		
6 to 15 years	24 (35.3%)	2 (4.4%)		
16 to 20 years	6 (8.8%)	2 (4.4%)		
21 or more years	19 (27.9%)	39 (86.7%)		
Practice for activity (hrs/week)				
< 5 h	22 (32.4%)	20 (44.4%)		
6 to 15 h	45 (66.2%)	18 (40%)		
16 to 20 h	0	2 (4.4%)		
21 or more hours	1 (1.5%)	5 (11.1%)		
Injury				
Yes	48 (69.6%)	12 (24%)	9 (14.1%)	9 (14.5%)
No	21 (30.4%)	38 (76%)	55 (85.9%)	53 (85.5%)
Chronic Disease				
Yes	11 (15.9%)	15 (30%)	43 (67.2%)	40 (64.5%)
No	58 (84.1%)	35 (70%)	21 (32.8%)	22 (35.5%)
Total (*N* = 245)	69	50	64	62

5 chess players did not report the number of years competed and hours/week practice in the activity

Masters Athletes and chess players were recruited whereas moderately active and inactive were acquired from the CCHS

The Kruskal-Wallis (K-W) tests indicated a statistically significant difference for rate of injury, *H*(3) = 73.2, *p* < 0.001, and prevalence of chronic disease among the activity groups, *H*(3) = 53.5, *p* < 0.001. Further exploration using a one-way ANCOVA indicated a significant effect for type of activity on the number of injuries reported, F(3, 236) = 28.39, *p* < 0.0001, partial η^2^ = 0.27, and observed power = 1.0 for *α* < 0.0125. Bonferroni post hoc tests indicated Masters Athletes were significantly different (*p <* 0.0001) than all activity groups, while no significant difference was found between chess players and the normative groups for incidence of injury (see Table 2). Interestingly, Chi-square analyses suggested a sprain or strain (52.1%) was the most common type of injury reported among injured Masters Athletes (*n* = 48), followed by multiple injuries (31.3%). In contrast, injured chess players (*n* = 12) primarily reported multiple injuries (41.7%) and/or a scrape or bruise (25%). Moderately active (*n* = 9) most commonly experienced a cut or puncture (33.3%) and/or a scrap or bruise (33.3%), whereas inactive older adults (*n* = 9) reported experiencing a broken or fractured bone (33.3%) and/or a sprain or strain (33.3%) (see Tables 2 and 3).

**Table 2:** Prevalence of injuries reported by athletes, chess players, moderately activity and inactive older adults

Activity Type	N	Mean (SD)	*p*
(A) Masters Athletes	69	1.46 (1.24)	AxB*, AxC*, AxD*
(B) Chess	50	0.58 (1.18)	AxB*
(C) Moderately activity	64	0.17 (0.46)	AxC*
(D) Inactive	62	0.16 (0.41)	AxD*
Total	245	0.62 (1.06)	

alpha adjusted ≤0.0125; SD: Standard Deviation; significance (*p*) is indicated by the asterisk (*)

**Table 3:** Prevalence of chronic diseases reported by athletes, chess players, moderately activity and inactive older adults

Activity Type	N	Mean (SD)	*p*
(A) Masters Athletes	69	0.20 (0.53)	AxC*, AxD*
(B) Chess	50	0.48 (0.84)	BxC*, BxD*
(C) Moderately activity	64	1.33 (1.27)	CxA*, CxB*
(D) Inactive	62	1.31 (1.46)	DxA*, DxB*
Total	245	0.83 (1.19)	

alpha adjusted ≤0.0125; SD: Standard Deviation; significance (*p*) is indicated by the asterisk (*)

Similarly, the chronic disease analyses indicated a significant effect for type of activity on the prevalence of chronic disease reported by older adults, F(3, 236) = 20.01, *p* < 0.0001, partial η^2^ = 0.20, and observed power = 1.0 for *α* < 0.0125. Interestingly, post hoc tests suggested Masters Athletes and chess players were not significantly different in the prevalence of chronic disease (*p* = 1.00), but were significantly different from both normative groups (*p <* 0.0001). In addition, no significant difference (*p =* 1.00) was found between the normative groups (Table 3). According to the Chi-square analyses, moderately active and inactive groups reported arthritis (29.7, 22.6%), high-blood pressure (28.1, 24.7%) and back problems (26.6, 25.8%) as the most prevalent types of chronic diseases, respectively. In contrast, chess players experienced, high-blood pressure (10%), back problems (8%) and diabetes (8%), while Masters Athletes commonly reported arthritis (5.8%) and high blood pressure (4.3%) (see Tables 4 and 5).

**Table 4:** Types of injuries experienced by athletes, chess players, moderately activity and inactive older adults

	Masters Athletes	Chess Players	Moderate Active	Inactive	Total Injured
Multiple Injuries	15 (31.3%)	5 (41.7%)	0	0	20 (25.6%)
Broken or fractured bones	0	0	1 (11.1%)	3 (33.3%)	4 (5.10%)
Sprain or Strain	25 (52%)	2 (16.7%)	0	3 (33.3%)	30 (38.5%)
Cut or puncture	0	0	3 (33.3%)	0	3 (3.8%)
Scrap, Bruise	3 (6.3%)	3 (25%)	3 (33.3%)	1 (11.1%)	10 (12.8%)
Other	5 (10.4%)	2 (16.7%)	2 (22.2%)	2 (22.2%)	11 (14.1%)
Total	48	12	9	9	78

Multiple injuries comprised of individuals with more than one type of injury

**Table 5:** Types of chronic diseases experienced by athletes, chess players, moderately activity and inactive older adults

	Masters Athletes	Chess players	Moderately active	Inactive
Asthma	2 (2.9%)	1 (2%)	4 (6.3%)	3 (4.8%)
Arthritis	4 (5.8%)	3 (6%)	19 (29.7%)	14 (22.6%)
Back problems	1 (1.4%)	4 (8%)	17 (26.6%)	16 (25.8%)
High-blood pressure	3 (4.3%)	5 (10%)	18 (28.1%)	17 (27.4%)
Migraine headaches	0	0	3 (4.7%)	5 (8.1%)
Chronic bronchitis	0	0	0	1 (1.6%)
Emphysema	0	0	0	2 (3.2%)
COPD	0	0	1 (1.6%)	3 (4.8%)
Diabetes	0	4 (8%)	7 (10.9%)	7 (11.3%)
Heart disease	1 (1.4%)	2 (4%)	3 (4.7%)	6 (9.7%)
Cancer	2 (2.9%)	0	1 (1.6%)	1 (1.6%)
Stomach or intestinal ulcers	0	0	2 (3.1%)	2 (3.2%)
Stroke	0	0	2 (3.1%)	1 (1.6%)
Urinary incontinence	0	2 (4%)	4 (6.3%)	1 (1.6%)
Bowel disorder	1 (1.4%)	3 (6%)	4 (6.3%)	2 (3.2%)
Total (*N* = 109)	11	15	43	40

Some participants reported prevalence of more than one type of chronic disease

*COPD* Chronic obstructive pulmonary disease

## Discussion

Advancing age is a unique constraint on health and function. It acts as both a risk factor for many health outcomes and a barrier to participation in physical or cognitive activities as well as other preventive behaviours such as medical physical examinations [52]. With this in mind, the current study compared injury and chronic disease rates among groups of older adults who maintained competitive involvement in a physically or cognitively challenging activity – namely, Masters Athletes and chess players. In addition, these two primary groups were compared to normative data from moderately active and inactive older adults [50].

In congruence with previous research [26, 49, 51], Masters Athletes reported significantly higher rate of injuries compared to all activity groups in the study; however, the partial η^2^ value (0.27) corresponded to a small effect size, perhaps due to the small samples [53]. Results from this study also reinforce findings from previous literature suggesting risk of injury can be higher in vigorous forms of physical activity such as competitive sport [32, 49]. Interestingly, the most common types of injuries among athletes were a sprain/strain and multiple injuries. In line with our findings, previous literature on Masters sport [32, 54, 55] suggests track and field athletes primarily experience minor orthopedic injuries in the lower extremities. Specifically, runners commonly sustain overuse injuries, knee pain, Achilles tendon issues, plantar fasciitis and shin splints [55]. In contrast, chess players primarily sustained multiple injuries and/or a scrape/bruise, while inactive older adults were found to be the only group to report broken bones.

In terms of chronic disease, type of activity significantly influenced the prevalence of chronic disease reported by older adults, although, once again, the effect size was small. Masters Athletes were significantly different to the normative groups on chronic disease prevalence, but not the chess players. Previous studies indicate sport participants have a significantly lower prevalence of chronic diseases compared to non-athletes [32, 56]. In addition to participation in often vigorous competition, lower prevalence of chronic disease could be contributed by additional lifestyle behaviors such as eating a healthy diet or limiting alcohol intake, cigarette smoking or sedentary time to a greater extent than the ‘average’ older adult [56]. As a result, increased participation in these various positive lifestyle behaviors may have improved the health outcomes of athletes.

Interestingly, chess players were also associated with a lower prevalence of chronic disease than the normative groups, even though increased sedentary time is recognized as an independent contributor for the decline in physical health [57]. Although chess is a sedentary activity, the competitive nature of the game can elicit certain physiological responses [47] that may encourage players to practice positive health behaviours (e.g., reduce smoking, healthy eating, regular medical examinations and positive expectations regarding aging) to advance in their level of competition [40, 52]. This is congruent with reports of chess grandmasters and championship competitors employing nutritionists and/or exercise trainers to prepare for the physical endurance required for matches [58]. Furthermore, similar to elite sport players, chess grandmasters are associated with a significantly greater life expectancy than the general population [40] suggesting competitive chess provides similar physical health benefits (i.e., mitigate chronic diseases) to competitive sport. In contrast, older adults who avoid cognitive engagement may find it challenging to manage not only cognitive and mental health [44–46, 59] but also physical health.

While considerable additional work is necessary to validate and replicate these results, they suggest competitive chess could be a viable option for older adults who are unable to participate in sport, since prevalence of a chronic disease was 3–4 times higher in the inactive group than Masters Athletes and chess players. Both normative groups reported increased prevalence of arthritis, high-blood pressure and back problems. Furthermore, future work is needed to determine whether these differences are due to participation in the activity itself (e.g., competition in a cognitively or physically challenging task) or to variables that might be related to these relatively unique groups (e.g., greater involvement in preventive health activities). These studies will extend our knowledge of the level and type of activity required to optimize health and function, as well as confirm whether participation in an intense cognitive activity alone can mitigate chronic disease amongst older adults. Given that chess players reported lower incident of injuries than Masters Athletes as well as a lower prevalence of chronic diseases compared to moderately active and inactive groups, it begs the question ‘why is the gold standard for successful aging only associated with those who participate in competitive sport?’ as suggested by Hawkins et al. [27]. Perhaps this label could be expanded to include other forms of competitive activity. Moreover, our lack of understanding regarding the consequences of competitive sport [24, 60], highlights the need to refrain from labeling Masters Athletes as the ideal model for successful aging, at least until additional work has been conducted. Furthermore, from a health promotion standpoint, advocating sport as a preventive health strategy may be problematic, particularly for individuals who have a) debilitating chronic diseases and/or socio-demographic barriers that can limit participation in sport [7, 61] or b) internalized the pervasive old age stereotypes in North American society and likely avoid participation in competitive sport [22]. Our study suggests older adults may have other options, such as competitive chess, to gain similar health benefits to sport participation. Moreover, it is possible older adults who participate in a variety of activities (e.g., chess *and* sport) may gain a combination of physical and cognitive health benefits [13, 62]. For that reason, advocating programs designed to cognitively and physically engage older persons may be a pragmatic response from both a health and an economic standpoint.

### Limitations

While this study had its strengths, by including a nationally representative dataset to generate normative data for group-based comparisons, as well as two unique activity groups, there were some limitations. For instance, Masters Athletes and chess players resided in the province of Ontario, whereas the normative groups were from Nova Scotia and British Columbia,^4^ which limits generalizations to other provinces or countries outside of Canada. Findings should also not be generalized to females, since all participants in this study were males. In addition, a cross-sectional study design limits our ability to test causal relationships between sport and chess participation and the physical health of older adults, since other genetic or environmental factors may be involved. Although not a limitation, this study did not compare ‘active’ older adults (i.e., the most active group in the CCHS) with Masters Athletes due to concerns about overlap between these groups. As a result, this limits what we can say about the value of high levels of sport participation compared to high levels of exercise involvement. It was also difficult to capture the nuances of socioeconomic status and its influence on the health of older adults, especially between the chess players and athletes, which would seem to be privileged groups. In addition, creating a more homogenous sub-sample may have concealed the effects of socio-demographic factors on the physical health of moderately active and inactive older adults, where the prevalence of chronic disease may have actually been higher among normative groups. Lastly, injured athletes and chess players who were unable to compete during the time of data collection may have led to an underestimation of injury and chronic disease.

## Conclusion

Older adults who are competitively active in sport and chess are able to mitigate the prevalence of chronic diseases compared to the general population. Interestingly, chess participation also resulted in a lower prevalence of injury. Therefore, for adults who are unable to be physically active, competitive chess may provide an alternative way to maintain both cognitive function and physical health, although the precise mechanisms of these effects are not clear.

## Footnotes

1 Researchers were limited to this cycle because injury and chronic disease questions used to recruit the primary groups were revised in the following cycles.

2 Sampling was limited to males because 100% of chess players were male.

3 EE (energy expenditure) = Frequency of the activity x Duration of activity (hours) x MET value (kcal.kg.hr)/365 days.

4 The CCHS includes many optional modules which health regions within provinces can opt out of according to their priorities.
